# Malaria among children under 10 years in 4 endemic health areas in Kisantu Health Zone: epidemiology and transmission

**DOI:** 10.1186/s12936-022-04415-z

**Published:** 2023-01-05

**Authors:** Gillon Ilombe, Junior Rika Matangila, Aimee Lulebo, Paulin Mutombo, Sylvie Linsuke, Vivi Maketa, Baby Mabanzila, Francis Wat’senga, Wim Van Bortel, Agossa Fiacre, Seth R. Irish, Pascal Lutumba, Jean-Pierre Van Geertruyden

**Affiliations:** 1 Unit of Entomology, Department of Parasitology, National Institute of Biomedical Research, Kinshasa, Democratic Republic of the Congo; 2 Unit of Clinical Pharmacology and Pharmacovigilance, Department of Base Science, Faculty of Medicine, University of Kinshasa, Kinshasa, Democratic Republic of the Congo; 3 Global Health Institute, Antwerp University, Antwerp, Belgium; 4 Department of Tropical Medicine, Faculty of Medicine, University of Kinshasa, Kinshasa, Democratic Republic of the Congo; 5 Faculty of Medicine, Public Health School, University of Kinshasa, Kinshasa, Democratic Republic of the Congo; 6 Department of Epidemiology Kinshasa, National Institute of Biomedical Research, Kinshasa, Democratic Republic of the Congo; 7 Unit of Entomology and Outbreak Research Team, Unit of Entomology, Institute of Tropical Medicine, Antwerp, Belgium; 8 PMI VectorLink Project, Abt Associates, Kinshasa, Democratic Republic of the Congo; 9 Division of Parasitic Diseases and Malaria, Center for Global Health, Centers for Disease Control and Prevention, President’s Malaria Initiative and Entomology Branch, Atlanta, GA USA

**Keywords:** Malaria infection, Determinants, Kisantu, Democratic Republic of the Congo

## Abstract

**Background:**

The Democratic Republic of the Congo (DRC) is the second most malaria-affected country in the world with 21,608,681 cases reported in 2019. The Kongo Central (KC) Province has a malaria annual incidence of 163 cases/per 1000 inhabitants which are close to the national average of 153.4/1000. However, the malaria prevalence varies both between and within health zones in this province. The main objective of this study was to describe the epidemiology and transmission of malaria among children aged 0 to 10 years in the 4 highest endemic health areas in Kisantu Health Zone (HZ) of KC in DRC.

**Methods:**

A community-based cross-sectional study was conducted from October to November 2017 using multi-stage sampling. A total of 30 villages in 4 health areas in Kisantu HZ were randomly selected. The prevalence of malaria was measured using a thick blood smear (TBS) and known predictors and associated outcomes were assessed. Data are described and association determinants of malaria infection were analysed.

**Results:**

A total of 1790 children between 0 and 10 years were included in 30 villages in 4 health areas of Kisantu HZ. The overall prevalence in the study area according to the TBS was 14.8% (95% CI: 13.8–16.6; range: 0–53). The mean sporozoite rate in the study area was 4.3% (95% CI: 2.6–6.6). The determination of *kdr-*west resistance alleles showed the presence of both L1014S and L1014F with 14.6% heterozygous L1014S/L1014F, 84.4% homozygous 1014F, and 1% homozygous 1014S. The risk factors associated with malaria infection were ground or wooden floors aOR: 15.8 (95% CI: 8.6–29.2), a moderate or severe underweight: 1.5 (1.1–2.3) and to be overweight: 1.9 (95% CI: 1.3–2.7).

**Conclusion:**

Malaria prevalence differed between villages and health areas within the same health zone. The control strategy activities must be oriented by the variety in the prevalence and transmission of malaria in different areas. The policy against malaria regarding long-lasting insecticidal nets should be based on the evidence of metabolic resistance.

**Supplementary Information:**

The online version contains supplementary material available at 10.1186/s12936-022-04415-z.

## Background

The Democratic Republic of the Congo (DRC) is, after Nigeria, the most affected malaria-endemic country with more than 29 million estimated cases annually [1]. The malaria control strategies in DRC are based on early diagnosis and prompt treatment, and vector control. There are three key vector control interventions in the National Strategic Plan: long-lasting insecticidal nets (LLINs), indoor residual spraying (IRS), and larval source management. The most widely implemented of these is the use of LLINs. LLINs are distributed for free through mass campaigns, school campaigns, and routine antenatal consultations [2]. According to the DRC National Malaria Control Programme (NMCP) 2016–2020 strategic plan, 55.8% of children under 5 years of age sleep under LLINs [2]. IRS is implemented by a small number of mining companies such as Tenke Fugurume Mining and some cement factories to protect their workers and neighboring communities [2]. Control activities through larval source management campaigns are not currently implemented at a large scale.

Vector control interventions are threatened by the emergence of insecticide resistance. In DRC, the primary vector, *Anopheles gambiae *sensu lato (*s.l*.) is generally susceptible to bendiocarb (carbamate) and pirimiphos-methyl (organophosphate), but resistant to DDT (organochlorine), permethrin, deltamethrin, and alpha-cypermethrin (pyrethroids) [3–7]. The impact of this resistance is undetermined, but in West Africa, resistance to pyrethroids resulted in a decrease in the efficacy of pyrethroid impregnated nets [8–10].

In DRC, the physical durability and effectiveness of nets impregnated with deltamethrin (DawaPlus 2.0) and α-Cypermethrin (Duranet) is only two years and not three years as recommended by the World Health Organization (WHO) [11]. Despite the frequent distribution of LLN during the past ten years, malaria transmission has remained stable in several areas of the DRC due, most probably for multiple reasons [12]. Amongst different reasons, the increased resistance against the insecticides used in the LLIN could play an important role [12]. Therefore, LLINs with other insecticides as synergistic mosquito nets was suggested [13]. A mixed entomological and epidemiological survey was conducted to identify the types of vectors and their resistance against some commonly used insecticides. At the same time, the disease transmission among the most vulnerable population groups was also clearly understood. These data will help to choose evidence-based and tailored interventions to successfully control malaria transmission.

## Methods

### Study area

This study was carried out in the four most malaria endemic health areas in Kisantu health zone (HZ) in Kongo Central (KC), one of 26 provinces of DRC. Kisantu is located in Lukaya district covering a surface area of 2400 km^2^ and a population of 153,188 inhabitants. In this area, the malaria incidence rate, in 2017, was 163/1000 Cases per person per year (DHIS2, 2017). The insecticide susceptibility testing conducted in the closest national entomological monitoring site, Kimpese (in the same province) in 2016 showed that *An. gambiae s.l.* was resistant to permethrin with 17% mosquito mortality in WHO susceptibility tests (95% CI: 11–26) and 76% to deltamethrin mortality (95% CI: 67–83)[14].

### Study design and study population

A mixed entomological and epidemiological study was carried out in 4 health areas of Kisantu health zone. The epidemiological study consisted of a stratified multistage cross-sectional study conducted in households with children aged 0 to 10 years. Informed and signed consent was obtained from the head of household. In case of illiteracy, fingerprint (thumb) consent in the presence of an independent witness was used.

The stratification took into account the model used in the strategic plan for the fight against malaria. This model divides the country in 3 zones according to the parasitic infection: hypo-endemic (<5%), holo-endemic (6–30%) and hyper-endemic zone (30%). The entomological study consisted of identifying species of *Anopheles* and their susceptibility to some insecticide.

#### Epidemiologic survey

##### Sample size

The number of children (n) included in the study was calculated using the following formula described by Kish (1965) for proportion [15].

$$\frac{{Z}_{\propto -\frac{1}{2}}^{2} . p . q}{{d}^{2}}\ge Deffx n$$

where p represents the estimated prevalence of malaria among children (p = 0.471), q = (1-p), the proportion of children under five years who get negative blood smear. (Demographic and Health Survey II, DRC)[16]; ${z}_{\propto -\frac{1}{2}}$, Z is the level of confidence based on the normal distribution. Z is 1.96l if the alpha is 0.05 in our calculation. d is the precision degree assumed at 5%; the design effect (deff) was assumed at 2. The minimal sample size computed was 765 children.

### Sampling

**Fig. 1:**
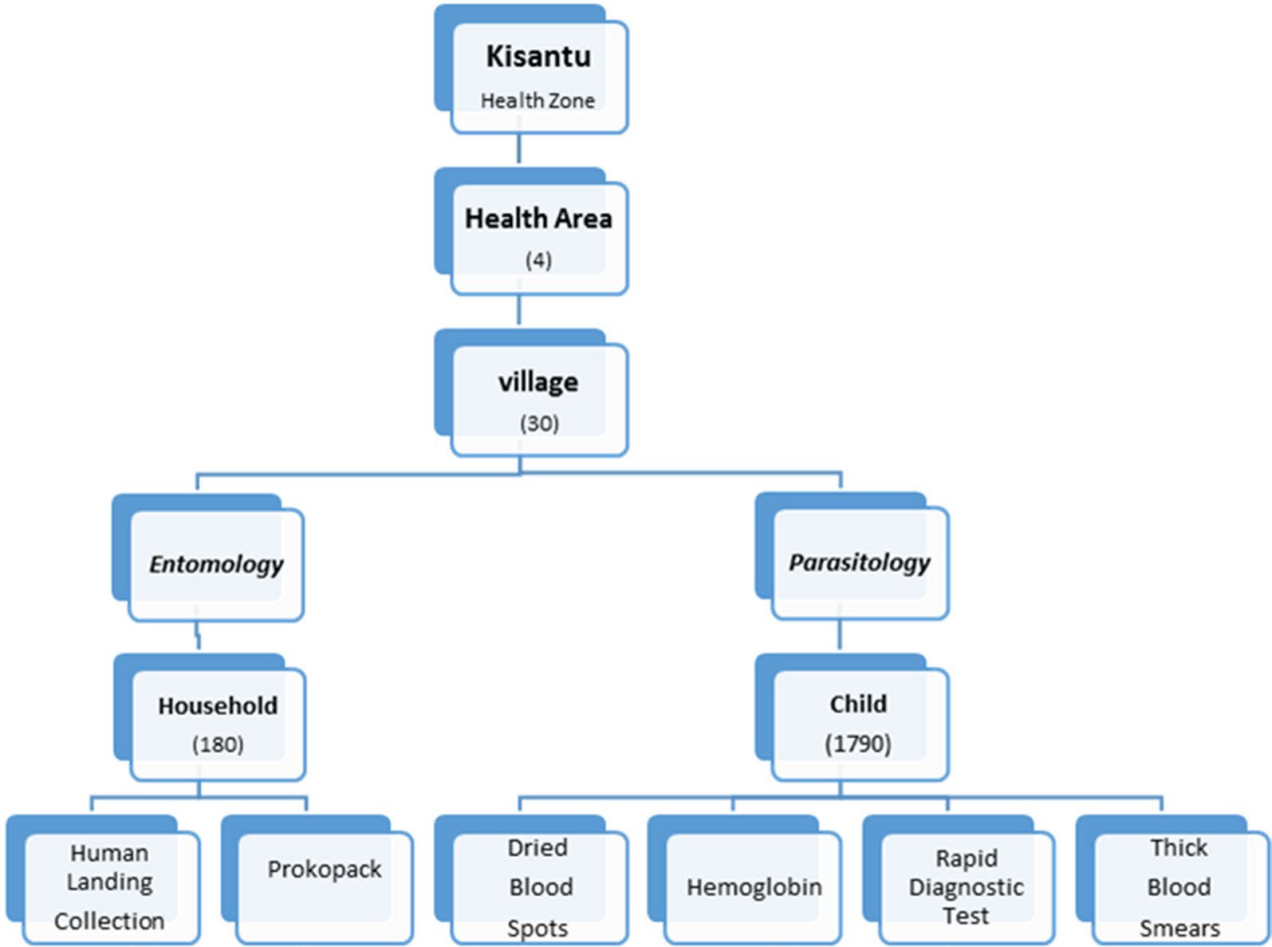
Design illustrating the entomological and epidemiological baseline data collection Kisantu 2018

A multi-stage sampling was used. In the first stage, 4 of the 15 Health areas within Kisantu Health Zone with the highest malaria prevalence were selected based on the 2017 parasitological surveys (Database of DLM 2017). In the second stage, 30 villages were selected randomly from the list of all the 72 villages in the four health areas using the Excel function *rand*. In the third stage, a census was conducted to collect a list of all children in these 30 villages between age 0 and 10 years included. A systematic approach was then used to identify children aged from 0 to 10 years per village preceded by the calculation of sampling interval by dividing the total number of eligible children in the villages by the sample size calculated as illustrated in Fig. 1.

### Data collection

A questionnaire was administered to each household of the selected children. Information on demographic characteristics (gender, age, literacy, occupation, and marital status of the parents/guardian), socio-economic status (of the parents/guardian) and related variables were collected. To assess the socio-economic level of the households, observations were made on household characteristics (i.e., material used for walls, roofs, and floors).

A thick blood smear (TBS) and a thin blood smear were taken from selected children in the households following WHO procedures [17]. Thick and thin smears were stained with 2% Giemsa, then stored in filing boxes and read with microscopes at the Clinic of the University of Kinshasa. When the thick smear was positive, a thin smear was used for the species determination and to assess the parasite density of the sample. The thick and thin smears were independently read by two readers. A third assessment was performed at the National Institute of Biomedical Research (NIRB) when a difference in TBS interpretation or a difference in parasite density in the thin blood smear of more than 15% was observed between the two readers. The result of the third reader who was the most experienced was taken into account. *Plasmodium* infection prevalence was calculated using the formula: total children with positive (*Plasmodium* infection) TBS / total children tested. The infection prevalence by village was used to group villages according to the DRC NMCP’s 2016–2020 strategic plans as: (i) hypo endemic ≤ 5%, (ii) meso -endemic 6–30% and (iii) hyper-endemic > 30% [2].

Haemoglobin levels were measured on the spot in the field using a portable automated HemoCue®Hb 301 haemoglobinometer system according to the manufacturer’s instructions. Anaemia was classified as mild, moderate, severe and life-threatening when haemoglobin concentrations were 9.0–9.9 g/dl, 7.0–8.9 g/dl, 5.0–6.9 g/dl and ˂5.0 g/dl, respectively [18].

The weight of children was measured using digital scales (Seca GmbH, Hamburg, and Germany) to the nearest 0.1 kg. The height was measured using a mobile measuring Seca mat (Seca GmbH, Hamburg, Germany) for children 0 to 99 cm and a recumbent length board for children of height > 99 cm to the nearest centimeter. The outcome variable was undernutrition indicated by wasting, stunting and underweight status in children >6 months of age. Stunting (height/age), wasting (weight/height) and underweight (weight/age) were defined as per WHO child growth standards. Children with Z scores less than two standard deviations (≤ 2 SD) below the median of the WHO child growth standards were classified as malnourished (stunted, wasted or underweight), minus 3 SD indicates severe stunting, wasting, and underweight[19]. Z scores of less than -2 SD were considered indicative of stunting [20].

### Entomological survey

Adult *Anopheles* were sampled from each of the same 30 villages in June 2018. Six households were randomly selected, of which human landing catches (HLC) were used in three of the houses for one night per house to estimate the biting rate. In the 3 other selected households, indoor resting mosquitoes were collected from the walls, roof and furniture using standard battery-powered Prokopack aspirators between 6:00 to 8:00 am. A total of 180 households were included in this survey (90 HLC, 90 aspirations).

The HLC collectors were placed indoor and outdoor with four collectors per household divided in two teams working six-hour shifts. The collectors were selected amongst the local population. After written informed consent, they were enrolled and trained on entomological activities. The collectors were referred to a health center and were treated free-of-charge if any illness occurred during the HLC activities and up to three weeks after collections.

The collected mosquitoes were morphologically identified [21] and subsequently stored in tubes with silica gel for later detection of sporozoites (CSP ELISA) and molecular analysis of alleles at the *Kdr* 1014 locus. Based on the biting rates and the *Plasmodium falciparum* prevalence, the *P. falciparum* entomological inoculation rate (PfEIR) was calculated the mean number of sporozoite-infected anopheles per house per night) and weighted to account for the proportion of collected anopheles processed for sporozoites. It is usually interpreted as the number of *P. falciparum* infective bites received by an individual during a night or season (aPfEIR) [22].

The sex and physiological status (fed, unfed, semi-gravid, and gravid) of the mosquitoes collected by Prokopack were determined. Subsequently they were dried on silica gel for the CSP by ELISA and the alleles of *kdr* 1014 locus, East and West together using the new developed technique from Huynh [23] at CDC Atlanta further analysis.

### Susceptibility testing

*Anopheles* larvae and pupae were collected in all villages included in the study and brought to the laboratory where they were reared to adults. Adult *Anopheles* species aged 3–5 days were tested using WHO susceptibility tube tests against the diagnostic concentrations of deltamethrin 0.05%; permethrin 0.75%; alpha-cypermethrin 0.05%; DDT 4%; bendiocarb 0.1% and PBO 4% following WHO guidelines [24]. After 24 h, mosquitoes were scored as dead or alive, identified morphologically and stored in tubes with silica gel for subsequent molecular analysis. Observed mortality was corrected using Abbott’s formula when control mortality varied between 5 and 20%. Experiments were repeated when control mortality was greater than 20% [25].

### Parasite infection detection and molecular analysis

*Anopheles gambiae s.l.* and *Anopheles funestus s.l.* heads and thoraces were separated from the abdomen and *P. falciparum* sporozoite antigen was detected using ELISA according to standard protocols [26]. ELISA tests were performed at the NIRB. DNA was extracted from legs and wings for PCRs to identify species and resistance mechanism (*kdr-w*). SINE PCR was used to identify the species of 200 mosquitoes morphologically identified as *An. gambiae s.l*. [27] *Kdr* resistance alleles 1014F were also determined by PCR on the same 200 mosquitoes using the protocol described by Huynh et al. [23]. The PCR assays were conducted at the Centers for Disease Control and Prevention (CDC) laboratory (Atlanta, GA, USA).

### Data analysis

Stata version 13.0 was used for statistical analysis. Descriptive statistics were used to summarize the characteristics of the study population. Continuous variables were reported using means with standard deviations when variables were normally distributed. If not, medians with ranges were reported. Categorical variables were summarized in frequencies or percentage. For the epidemiological study, the prevalence was calculated using all children who had a positive thick drop, over the total study population.

χ^2^ test was used to search for the association between strata of malaria transmission and some characteristics of the households (gender, age, weight, size, temperature, and number of people living in the household, number of sleeping spaces, occupation and level of education of the head of household, house type and nutritional status). Statistically significant associations were thereafter assessed in a logistic regression analysis to determine factors independently associated with malaria transmission. To take in account the cluster, the vce (cluster clustvar) option was used. The odds ratio (OR) with a corresponding 95% confidence interval was reported to quantify the strength of association. Significance was set at p-value of 0.05. Mapping of vector distribution and prevalence was also performed. Student’s t test was used to compare means of dead anopheles according to the insecticide.

The following formulas were used to calculate entomological indicators:

- The sporozoite rate (SR) = (ELISA positive total number/ total number of *Anopheles* species tested) × 100
- Human biting rate (HBR) = total number of *Anopheles* species collected by HLC during a specific period / total number of man-nights
- Nightly entomological inoculation rate (EIR) = Nightly HBR × SR.

## Results

### Study participants characteristics

A total of 1790 children were included in this study in 30 villages in the Kisantu health zone. Nearly half of the children were female (51.4%) or aged under five years (51.7%) (Table 1).

**Table 1:** General characteristics of the study population in the 4 areas in Kisantu Health Zone, Kongo-Central Province, 2018

Variables	n
Number of participants	1790
Demographic variables	
Male (n (%; 95% CI)	870 (48.6; 49.1–43.7)
Age (year; mean, SD)	5.1 ± 3.1
Age < 5 years (n (%; 95% CI)	925(51,7; 49.3–54.1)
Hb (gr/l) Mean ± SD	9.9 ± 1.7
Weight (kg) Mean ± SD	15.5 ± 5.8
Height (cm) Mean ± SD	100.5 ± 20.8
Temperature (°C) Mean ± SD	36.7 ± 0.5
Socio-economic status of the household	
Number of persons per household (Mean ± SD)	7.7 ± 2.8
Number of children below 10 years (Mean ± SD)	3.5 ± 1.5
Number of sleeping space (Mean ± SD)	2.8 ± 0.9
Profession of household head (n (%; 95% CI)	
Jobless	193 (10.8; 9.3–12.4)
Farming/fishing/hunting/animal rearer	1154 (64.5; 60.8–68.3)
Civil servant	197 (11.0; 9.5–12.6)
Independent	151 (8.4; 7.2–9.2)
Other	95 (5.3; 4.3–6.4)
Educational level of household head	
Unschooled	76 (4.3; 3.5–5.4)
Primary	1201 (68.8; 66.2–70.6)
Secondary	410 (23.4; 21.4–25.4)
Institute/University	67 (3.8; 2.9–4.9)
Main floor type in the house	
Cement/tiles	687(38.4; 36.1–40.7)
Ground/Bamboo	1103(61.6; 59.3–63.8)
Main external wall types	
Cement blocks	142(7.93; 6.7–9.3)
Mud blocks	1251(69.9; 66.1–73.8)
Mud plaster on wooden support	350(19.5; 17.6–21.7)
Mud walls /Straw	47(2.6; 1.9–3.5)
Main roofing material	
Zinc	791(44.2; 41.2–47.3)
Straw	741(41.4; 38.5–44.5)
Thatch	258(14.4; 12.7–16.2)
Nutritional*	
Stunting	
Normal/mild	967(54.0; 50.7–57.5)
Moderate/severe	608(34.0; 31.4–36.7)
Over	215(12; 10.5–13.7)
Wasting	
Normal/mild	1211(67.7; 63.9–71.5)
Moderate/severe	268(15; 13.3–16.8)
Over	215(12; 10.5–13.7)
Underweight	
Normal/mild	1050(58.7; 55.2–62.3)
Moderate/severe	461(25.8; 23.5–28.2)
Over	279(15.6; 13.8–17)
Malaria infection	
TBS positive	266(14.8; 13.3–16.6)
*Plasmodium falciparum*	244(93.5; 89.9–96.0)
*Plasmodium malariae*	10(3.8; 1.9–6.7)
*Plasmodium falciparum* + *malariae*	2(0.8; 0.1–2.5)
*Gametocyte Plasmodium falciparum*	5(1.9; 0.7–4.2)

^*^100 children were brought back to the cause of age < 6 months

Socio-economically, 64.5% of heads of households were farmers, fishermen or cattle breeders, and the majority had a primary school education level. The great majority (69.9%) of houses visited was built with mud walls. The most frequent roofing material was steel sheeting (44.2%).

The averages of the weight, height, and temperature of the children were 15.5 ± 5.8 kg; 100.5 ± 20.5 cm, and 36.7 ± 0.5 °C, respectively (Table 1). Regarding malnutrition, three types were observed in the children surveyed: 34% showed signs of delay of growth, 15% had insufficient weight and 25.8% had a moderate or severe malnutrition. The median of the haemoglobin was 9.9 ± 1.7 g/dl (Table 1).

### *Plasmodium* infection prevalence

The overall prevalence of *Plasmodium* infection according to the TBS was 14.8% (95CI:13.3–16.6) (Table 1). Fifteen of the villages were classified as hypo-endemic [Nenga-kimasi and Kundulu, respectively of 36.7% (95% CI 24.6–50.1) and 50.9% (95% CI 37.5–64.1)], twelve as meso-endemic [Luango and Nenga-groupement, respectively of 20.3% (95% CI 10.9–38.8) and 28.3% (95% CI 17.5–41.4)] and three as hyper-endemic [Kingombi and Kikola, respectively of 3.4% (95% CI 0.4–11.7) and 1.7% (95% CI 0.0–8.9)] (Fig. 3). *Plasmodium falciparum* was the primary *Plasmodium* species 93.5% (95% CI 89.9–96.0). *Plasmodium malariae* represented 3.8% (CI 1.9–6.7 and 0.77% (CI 0.1–2.5) were mixed infections *P. falciparum* and *P. malariae*.

Main type of wall, the type of ground, the main type of domestic roof (straw and thatch) and malnutrition were associated with *Plasmodium* infection. In multi-variate analysis, having ground or wood floors (aOR: 2.8; CI95%: 2.1–3.4) and having moderate or severe malnutrition (aOR: 1.6; CI95%:1.1–3.5) were associated to the emergence of *Plasmodium* infection (Table 2).

**Table 2:** Bivariate and multivariate analysis determinants of epidemiological stratum (zone hypo/meso endemic and hyper endemic)

Variables	Bivariate analysis Crude OR (95% CI)	p value	Multivariate analysis Adjusted OR (95% CI)	p value
Wall type				
Mud walls/Straw	2.1(1.5–2.8)	**0.001**		
Cement block	1			
Floor type				
Ground/wood	15.7 (8.5–32.2)	**0.001**	15.8 (8.6–29.2)	** < 0.001**
Cement/tiles	1			
Roofing type				
Straw	2.7(1.9–3.8)	**0.001**		
Thatch/wood	4.5(3.0–6.7)	**0.001**		
Zinc	1			
Wasting				
Moderate/severe	1.5 (1.1–2.2)	**0.024**	1.5 (1.1–2.3)	0.025
Over	1.9 (1.3–2.6)	**0.001**	1.9 (1.3–2.7)	** < 0.001**
Normal/mild	1			
Stunting				
Moderate/severe	1.5 (1.1–1.9)	**0.010**		
Over	1.3 (0.9–2.0)	**0.198**		
Normal/mild	1			
Underweight				
Moderate/severe	1.3 (1.0–1.8)	**0.071**		
Over	1.1 (0.7–1.6)	**0.748**		
Normal/mild	1			

Given the high collinearity between independent variables such as types of soil, roofing and walls, only the type of soil was taken into account in the model. And for nutritional status because of the multicollinearity between stunting, wasting and underweight, only wasting is included in the model

The odds ratio (OR) with a corresponding 95% confidence interval was reported to quantify the strength of association. Bold values indicate statistically significant p values (p < 0.05)

### Entomological survey results

#### *Anopheles* mosquito composition, behaviour and human biting rate

**Fig. 2:**
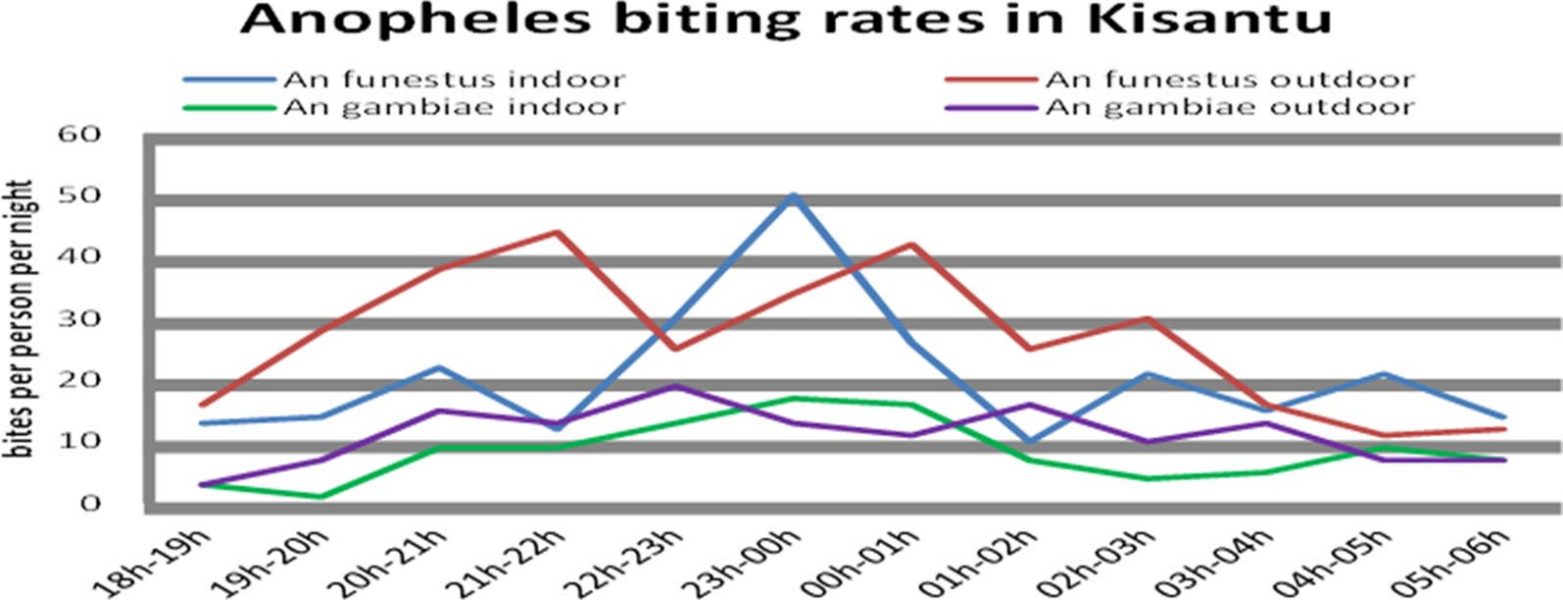
Assessment of *Anopheles funestus* and *An. gambiae s.l.* activity based on the human landing collection, Kisantu 2018

**Fig. 3:**
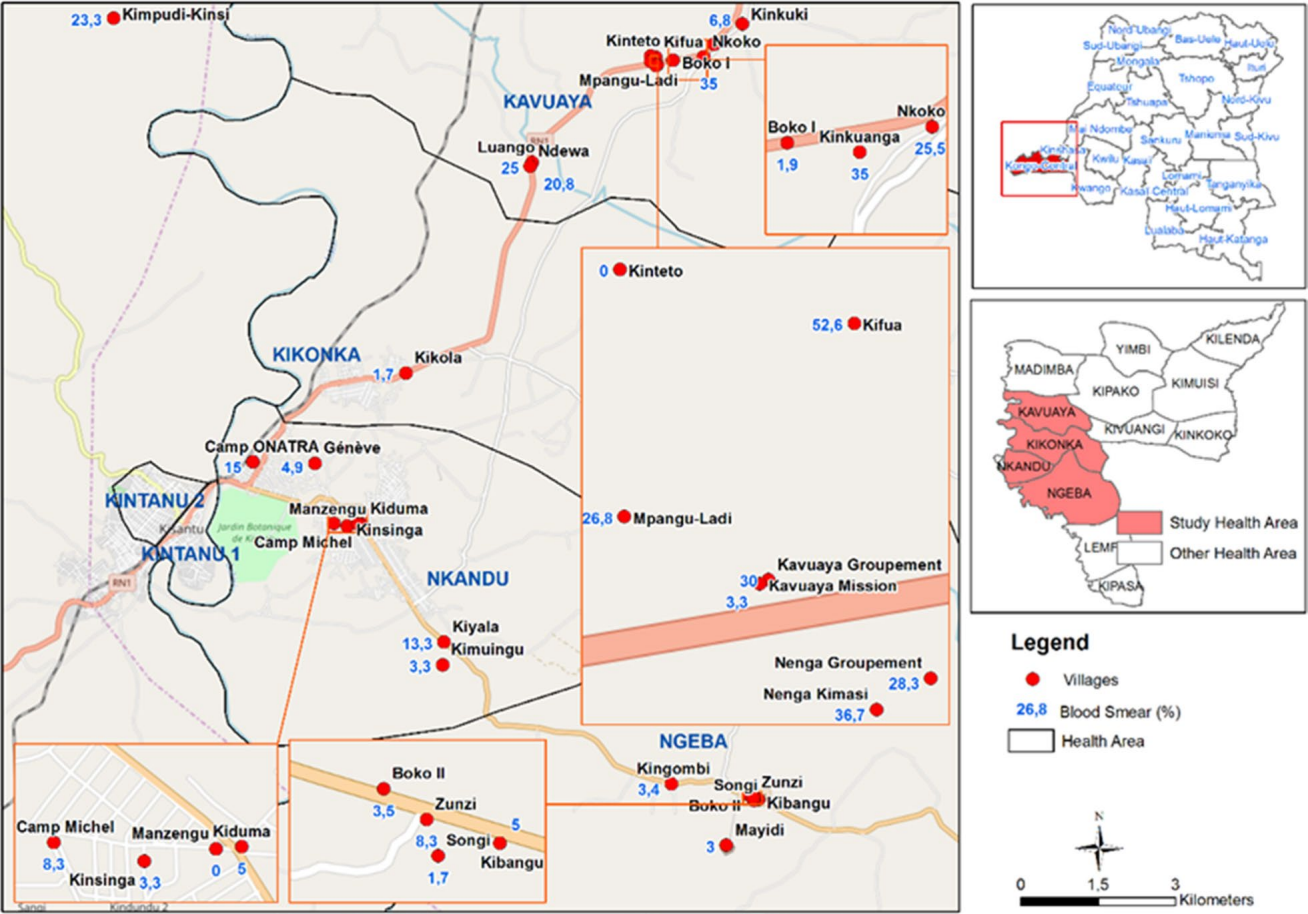
Local variation of malaria infection prevalence by villages in the 4 areas in Kisantu Health Zone, Kongo-Central Province

*Anopheles funestus s.l.* was the most abundant 58.2% (CI 95%: 55.1–61.3) species collected followed by the *Anopheles gambiae s.l*. 24.1% (CI 95%: 21.5–26.9) (Table 3). Other *Anopheles* species were, respectively, 12.8% (CI 95%: 10.9–15.0) for *Anopheles nili,* 1.8% (CI 95%: 1.1–2.9) for *Anopheles paludis and* 3.0% (CI 95%: 2.0–4.2) for *Anopheles coustani*. A total of 1049 Culicinae were also captured. Both *An. gambiae s.l*. and *An. funestus s.l.* indoor biting behaviors were like those of outdoor biting (Fig. 2). They were active between 6:00 pm and 6:00 am. The maximal mean peak of *An. funestus s.l* of 44 bites per person per night between 21:00 and 22:00 outdoor and of 50 bites per person per night between 23:00 pm and 0:00 am indoor (Fig. 3).

**Table 3:** The number of specimens collected by species of Anopheles in the 4 areas in Kisantu Health Zone

Mosquitoes	HLC		Aspiration	Total (%)
	In	Out		
*An. funestus s.l*	234 (23.7)	303 (30.6)	35 (3.5)	572 (58.2)
*An. gambiae s.l*	96 (9.7)	134 (13.5)	7 (0.7)	237 (24.1)
*An. coustani*	10 (1.0)	19 (1.9)	0	29 (3.0)
*An. nili*	52 (5.3)	74 (7.5)	0	126 (12.8)
*An. paludis*	7 (0.7)	11 (1.1)	0	18 (1.8)
Total				982 (100)

The highest human biting rates were recorded in the villages of Boko I, Kifua and Kinkuanga with rates of 11.6, 21.3 and 9.6 bites per person per night, respectively. Overall, the mean sporozoite index in the Health Zone of Kisantu was 4.3% (95% CI: 2.6–6.6), (Table 4).

**Table 4:** Mortality of *An. gambiae* in WHO tube tests with insecticides Entomological Inoculation Rate (HLC Indoors & Outdoors) in the 4 areas in Kisantu Health Zone

Entomological indicator	n (%; 95% CI)	Mean difference in mortality	P value
Insecticide susceptibility of *An. gambiae s.l*			
Permethrin 0.75%	100(25; 16.5–33.5)	58	< 0.001
Permethrin 0.75% + PBO	100(83; 75.6–90.4)		
Deltamethrin 0.05%	100(62; 52.5–71.5)	37	< 0.001
Deltamethrin 0.05% + PBO	100(99; 97.1–100)		
a-Cypermethrin	100(48; 38.2–57,8)	44	< 0.001
a-Cypermethrin + PBO	100(92; 86.7–97.3)		
DDT 4%	100(11; 4.9–17.1)	18	< 0.0015
DDT 4% + PBO	100(29; 20.1–37.9)		
Bendiocarb	100(100; 98–100)	NA	NA
Plasmodium falciparum circumsporozoite			0.621
*An. gambiae s.l*	200(3.5; 1,5–6,8)		
*An. funestus s.l*	200(5; 2.6–8.7)		
Total	400(4.3; 2.6–6.6)		
Human biting rate (p/night/p/person)	n		
*An. gambiae s.l*	1.27		
*An. funestus s.l*	2.98		
total	4.26		
Nightly entomological inoculation rate (p/night)	n		
*An. gambiae s.l*	0.04		
*An. funestus s.l*	0.15		
Total	0.18		

The entomological inoculation rate (EIR) per night was estimated at 0.04 infectious bites per night for *An. gambiae s.l.* and 0.15 for *An. funestus s.l.* and one person could receive from both vectors 0.18 infectious bites per night.

#### Insecticide resistance profile and molecular analysis results

The mosquito populations tested were resistant with insect mortality rates to permethrin of 25% (95% CI: 17–34), deltamethrin of 62% (95% CI:53–72%), alpha-cypermethrin of 48% (95% CI: 38–58), DDT of 11% (95% CI:5–17%) and fully susceptible to bendiocarb (100%) (Table 4). However, results obtained with PBO pre-exposure had higher mortality rates for: permethrin 83% (95% CI: 76–90), deltamethrin 99% (95% CI: 97–100%), alpha-cypermethrin 92% (95% CI: 87–97), DDT 29% (95% CI: 20–38).

The PCR results showed that all *An. gambiae s.l*. mosquitoes analysed for the species identification were *An. gambiae *sensu stricto (*s.s*.). In those samples, 14.6% are heterozygous (*kdr-east (L1014S) /kdr-west (L1014F))*, 84.4% are homozygotes *kdr-west (L1014F)*, and ~ 1% are *kdr-east (L1014S)*. The allele frequency was 0.99 for *kdr-west* and 0.16 for *kdr west–east*. (Table 5).

**Table 5:** Genotypes and allele frequency of the 1014F and 1014S *kdr* mutation in the Kisantu Health Zone

*kdr*	Number tested	Proportion (%)	Genotype				F(kdr)
			SS	RR	RS	Did not amplify	
*kdr-west(L1014F)*	200	84.4	2	197	0	1	0.99
*kdr-east(L1014S)*	200	1	168	31	0	1	0.16
*Kdr –west/east*	200	14.6					

*RR* homozygous resistant, *SS* homozygous sensitive, *RS* Heterozygote resistant, *F (kdr)* allele frequency of resistant *kdr* gene

## Discussion

*Plasmodium* baseline prevalence found by TBS was 14.8% in children between 0 and 10 years old. This study showed disparities in infection prevalence between villages in the same health zone. The heterogeneity in malaria prevalence was expected, as malaria prevalence varies with a number of epidemiological and environmental factors, including land use, soil type and hydrography [28, 29]. The National Malaria Control Program should take into account this heterogeneity of prevalence by a consolidated target of high-quality service coverage in the fight against malaria. This should be in health facilities and in the community in terms of prevention, both individual and collective, care, epidemiological surveillance and control of outbreaks. Further, it was observed that living in a house with ground floor and suffering from malnutrition are correlated with a risk factor for *Plasmodium* stratum epidemiological infection.

The sporozoite rate found in this study is similar to that reported in other studies [30]. From the species point of view no difference was found between *An. gambiae s.l* and *An. funestus s.l*. Both *An. gambiae s.l.* and *An. funestus s.l.,* were found in the study site with a predominance of *An. funestus* s*.l.* [3]. The presence of several rivers and swamps, typical ecology for *An. funestus s.l* might explain the predominance of this species in Kisantu. Similar results were observed in Kinshasa [5] and elsewhere in the country including Kongo Central [30]. *Anopheles gambiae s.l*. and *An. funestus s.l.* had a mean biting peak indoor and outdoor between 22:00 and 23:00 and 23:00–00:00am, respectively. The similar bite peak was observed in Pawa village (Haut Uele) sentinel site 2017 through the President Malaria Initiative (PMI) entomological monitoring activities [30]. The EIR was estimated at 1.2 per person per night, for *An. gambiae s.l.* and 4.5 per person per night for *An. funestus s.l.*

The Phenotypic resistance to pyrethroids was observed in *An. gambiae s.l.* populations. This resistance to pyrethroids could have a negative impact on the efficacy of LLINs impregnated with pyrethroid [3, 5]. Susceptibility was noted with bendiocarb, which could open the possibility for controlling the pyrethroid resistant mosquitoes through indoor residual spraying. A recovery of deltamethrin susceptibility of *An. gambiae s.l.* observed after pre-exposure to PBO showed the likely implication of metabolic resistance mediated by oxidases [5]. A genetic resistance against commonly used insecticides in LLIN, predominantly in the east, was showed in this study. This situation could maintain an important transmission and calls for the current strategy to be reviewed. These results are similar to those found by some authors at the Nord Ubangi site in DRC showing the high frequency of 1014F in *An gambiae s.s.* populations [31].

Malaria control strategy in DRC is based on detection, treatment and prevention. The prevention is based mainly on intermittent preventive treatment (IPT) and LLINs. The observed resistance of *An. gambiae s.s.* could have a negative impact in LLINs efficacy [32]. The resistance level of the vectors should be carefully surveyed but also the development of an alternative strategy such as the deployment of LLINs combining with PBO. The study noticed that 33% of children were malnourished with a risk of developing malaria infection. These results are like those found in Kinshasa by Maketa et al*.* The authors found that malnourished children were almost twice as likely to develop malaria compared to healthy children [19, 33]. The present study has some limitations. First, this is a cross sectional study which does not have as goal to confirm an effect-cause relationship between malaria prevalence and associated factors found but to provide snapshots of the situation. Second, the ICC used was of 0.02 [34] and a design effect of 2 for the sample size calculation due to the lack of a previous similar study conducted in the same setting. With the data found, the ICC was of 0.08 while the design effect should have been around 6; the study acknowledges that it did not have sufficient power.

## Conclusion

In these selected Health areas, malaria infection prevalence was high in some villages compared to the other villages in the Kisantu health zone. House type was highlighted as a risk factor of having malaria infection. Malnutrition was associated with malaria endemicity. Malaria transmission vector is mostly *An. funestus s.l* and *An. gambiae s.l*. These vectors were resistant to pyrethroids, but more susceptible when PBO was used in pre-exposure phase. The policy against malaria regarding LLINs should be based on the evidence of metabolic resistance and the biting period including the peak which is around 23h00.

## Supplementary Information

**Additional file 1. Figure S4.** Mapping of distribution of Anopheles species in Kisantu Health Zone, Kongo-Central Province. **Table S6.** Human biting rate was determined from human landing collections in the different villages in Kisantu only *An. gambiae sl* and *funestus sl*. **Table S7. **Study sites and their epidemiological stratum in Kisantu Health Zone, Kongo-Central Province.

## Data Availability

All relevant data supporting the findings of this study are included within the paper and their Additional file 1.

## Abbreviations

DRC: The Democratic Republic of the Congo
LLINs: Long-lasting insecticidal nets
IRS: Indoor residual spraying
NMCP: National Malaria Control Program
PBO: Piperonyl butoxide
HZ: Health zone
KC: Kongo Central
TBS: Thick blood smear
HLC: Human landing catches
SR: Sporozoite rate
HBR: Human biting rate
EIR: Nightly entomological inoculation rate
