# Supplementary material for: Malaria among children under 10 years in 4 endemic health areas in Kisantu Health Zone: epidemiology and transmission
Source: Malar J. 2023 Jan 5;22:3. doi: 10.1186/s12936-022-04415-z (PMC9814333; doi:10.1186/s12936-022-04415-z)
Supplement: Supplementary file 1 — Additional file 1. Figure S4. Mapping of distribution of Anopheles species in Kisantu Health Zone, Kongo-Central Province. Table S6. Human biting rate was determined from human landing collections in the different villages in Kisantu only An. gambiae sl and funestus sl. Table S7. Study sites and their epidemiological stratum in Kisantu Health Zone, Kongo-Central Province. [file 12936_2022_4415_MOESM1_ESM.docx]

**Malaria among children under 10 years in 4 endemic health areas in Kisantu Health Zone: epidemiology and transmission**

**Additional**

*
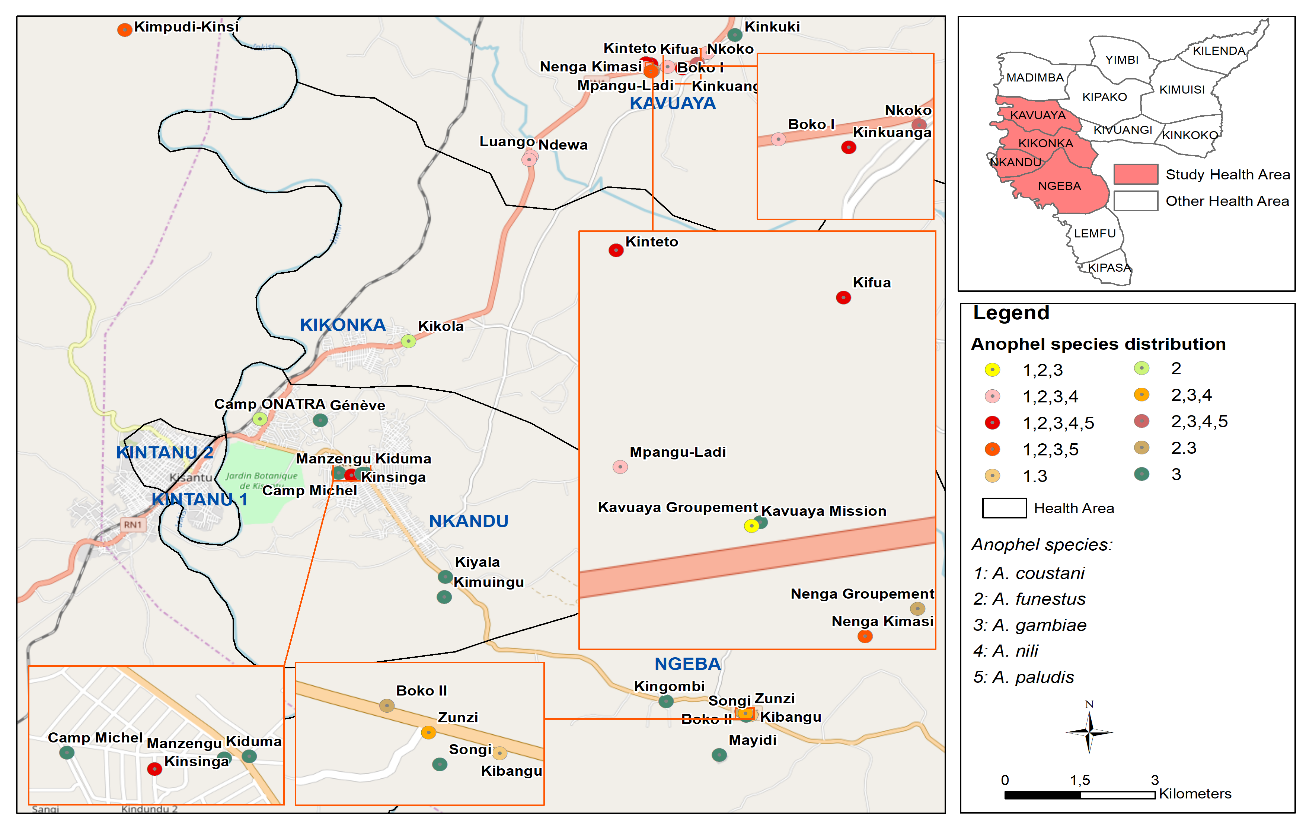
*

*Figure 4: Mapping of distribution of Anopheles species in Kisantu Health Zone, Kongo-Central Province*.

Table 6. Human biting rate was determined from human landing collections in the different villages in Kisantu only *An. gambiae sl* and *funestus sl*

| **Village** | **Mosquitoes** | | | **N**  **HLC**  **In/Out** | **Bites per man-night** | | |
| --- | --- | --- | --- | --- | --- | --- | --- |
|  | All | In | out |  | Inside | Outside | Mean HBR |
| Kifua | 128 | 55 | 73 | 3 | 18.3 | 24.3 | 21.3 |
| Ndewa | 110 | 58 | 52 | 3 | 19.3 | 17.3 | 18.3 |
| Kinteto | 91 | 24 | 67 | 3 | 8 | 22.3 | 15.2 |
| Luango | 87 | 35 | 52 | 3 | 11.6 | 17.3 | 14.5 |
| Nkoko | 82 | 40 | 42 | 3 | 13.3 | 14 | 13.6 |
| Boko I | 69 | 27 | 42 | 3 | 9 | 14 | 11.5 |
| Mpangu-Ladi | 68 | 29 | 39 | 3 | 9.6 | 13 | 11.3 |
| Zunzi | 61 | 24 | 37 | 3 | 8 | 12.3 | 10.2 |
| Kinkuanga | 58 | 36 | 22 | 3 | 12 | 7.3 | 9.6 |
| Kundulu | 51 | 20 | 31 | 3 | 6.6 | 10.3 | 8.5 |
| Kavuaya Mission | 47 | 20 | 27 | 3 | 6.6 | 9 | 7.8 |
| Kinsinga | 39 | 25 | 14 | 3 | 8.3 | 4.6 | 6.5 |
| Nenga Kimasi | 37 | 13 | 24 | 3 | 4.3 | 8 | 6.2 |
| Nenga Groupement | 36 | 14 | 22 | 3 | 4.6 | 7.3 | 6 |
| Kimpudi-Kinsi | 16 | 0 | 16 | 3 | 0 | 5.3 | 2.6 |
| Boko II | 3 | 2 | 1 | 3 | 0.6 | 0.3 | 0.5 |
| Camp Michel | 0 | 0 | 0 | 3 | 0 | 0 | 0 |
| Camp Onatra | 2 | 1 | 1 | 3 | 0.3 | 0.3 | 0.3 |
| Geneve | 2 | 1 | 1 | 3 | 0.3 | 0.3 | 0.3 |
| Kavuaya groupement | 2 | 1 | 1 | 3 | 0.3 | 0.3 | 0.3 |
| Kibangu | 2 | 1 | 1 | 3 | 0.3 | 0.3 | 0.3 |
| Kiduma | 0 | 0 | 0 | 3 | 0 | 0 | 0 |
| KIkola | 2 | 1 | 1 | 3 | 0.3 | 0.3 | 0.3 |
| Kimuingu | 2 | 1 | 1 | 3 | 0.3 | 0.3 | 0.3 |
| Kingombi | 2 | 1 | 1 | 2 | 0.3 | 0.3 | 0.3 |
| Kinkuki | 2 | 1 | 1 | 3 | 0.3 | 0.3 | 0.3 |
| Kiyala | 1 | 0 | 1 | 3 | 0 | 0.3 | 0.2 |
| Manzengu | 2 | 1 | 1 | 3 | 0.3 | 0.3 | 0.3 |
| Mayidi | 2 | 1 | 1 | 3 | 0.3 | 0.3 | 0.3 |
| Songi | 2 | 1 | 1 | 3 | 0.3 | 0.3 | 0.3 |
|  |  |  |  |  |  |  |  |

Table 7. Study sites and their epidemiological stratum in Kisantu Health Zone, Kongo-Central Province

| **Villages** | **Geographical coordinates** | | **Altitude** | **n** | **Prevalence (95% CI)** | **Stratum Epidemiological** |
| --- | --- | --- | --- | --- | --- | --- |
|  | **South** | **East** |  |  |  |  |
| Boko I | 05°03.281’ | 015°08.703’ | 515m | 59 | 1.7 (0.0-9.1) | Hypo-endemic |
| Boko Ii | 05°11.005’ | 015°09.507’ | 470m | 58 | 3.5 (0.4-11.9) | Hypo-endemic |
| Camp Michel | 05°08.147’ | 015°05.150’ | 470m | 60 | 5 (1.0-13.9) | Hypo-endemic |
| Camp Onatra | 05°07.502’ | 015°04.299’ | 470m | 60 | 8.3 (2.7-18.4) | Meso-endemic |
| Geneve | 05°07.516’ | 015°04.951’ | 470m | 60 | 15 (7.1-26.6) | Meso-endemic |
| Kavuaya Groupement | 05°03.306’ | 015°08.501’ | 515m | 60 | 30 (18.8-43.2) | Meso-endemic |
| Kavuaya Mission | 05°03.307’ | 015°08.499’ | 515m | 61 | 4.9 (1.0-13.7) | Hypo-endemic |
| Kibangu | 05°11.048’ | 015°09.599’ | 631m | 60 | 3.3 (0.4-11.5) | Hypo-endemic |
| Kiduma | 05°08.154’ | 015°05.441’ | 470m | 61 | 4.9 (1.0-13.7) | Hypo-endemic |
| Kifua | 05°03.249’ | 015°08.520’ | 515m | 57 | 52 (38.9-66.0) | Hyper-endemic |
| Kikola | 05°06.568’ | 015°05.904’ | 470m | 60 | 1.7 (0.0-8.9) | Hypo-endemic |
| Kimpudi-Kinsi | 05°02.840’ | 05°02.840’ | 515m | 60 | 23.3 (13.4-36.0) | Meso-endemic |
| Kimuingu | 05°09.364’ | 015°06.289’ | 470m | 60 | 3.3 (0.4-11.5) | Hypo-endemic |
| Kingombi | 05°10.886’ | 015°08.685’ | 470m | 59 | 3.4 (0.4-11.7) | Hypo-endemic |
| Kinkuanga | 05°03.302’ | 015°08.864’ | 515m | 60 | 35 (23.1-48.4) | Hyper-endemic |
| Kinkuki | 05°02.900’ | 015°09.431’ | 515m | 59 | 6.9 (1.9-16.5) | Meso-endemic |
| Kinsinga | 05°08.176’ | 015°05.290’ | 470m | 60 | 3.3 (0.4-11.5) | Hypo-endemic |
| Kinteto | 05°03.237’ | 015°08.468’ | 515m | 60 | 0.0 (0.0-5.9) | Hypo-endemic |
| Kiyala | 05°09.395’ | 015°06.302’ | 470m | 60 | 13.3 (5.9-24.6) | Meso-endemic |
| Kundulu | 05°03.111’ | 015°09.131’ | 515m | 59 | 50.9 (37.5-64.1) | Hyper-endemic |
| Luango | 05°04.354’ | 015°07.227’ | 515m | 59 | 20.3 (10.9-38.8) | Meso-endemic |
| Manzengu | 05°08.157’ | 015°05.401’ | 470m | 60 | 0.0 (5.9) | Hypo-endemic |
| Mayidi | 05°11.529’ | 015°09.261’ | 631m | 60 | 3.3 (3.4-11.5) | Hypo-endemic |
| Mpangu-Ladi | 05°03.292’ | 015°08.469’ | 515m | 60 | 26.7 (16.1-34.7) | Meso-endemic |
| Ndewa | 05°04.394’ | 015°07.205’ | 515m | 61 | 24.6 (14.5-37.3) | Meso-endemic |
| Nenga Groupement | 05°03.328’ | 015°08.537’ | 515m | 60 | 28.3 (17.5-41.4) | Meso-endemic |
| Nenga Kimasi | 05°03.335’ | 015°08.525’ | 515m | 60 | 36.7 (24.6-50.1) | Hyper-endemic |
| Nkoko | 05°03.245’ | 015°09.025’ | 515m | 57 | 28.1 (16.9-41.5) | Meso-endemic |
| Zunzi | 05°11.029’ | 015°09.541’ | 631m | 60 | 8.3 (2.7-18.4) | Meso-endemic |
| Songi | 05°11.058’ | 015°09.550’ | 631m | 60 | 1.7 (0.0-8.9) | Hypo-endemic |
